# Circuit imaging biomarkers in preclinical and prodromal Parkinson's disease

**DOI:** 10.1186/s10020-021-00327-x

**Published:** 2021-09-16

**Authors:** Sanne K. Meles, Wolfgang H. Oertel, Klaus L. Leenders

**Affiliations:** 1 Department of Neurology, University Medical Center Groningen, University of Groningen, Hanzeplein 1, PO Box 30.001, 9700 RB Groningen, The Netherlands; 2 Department of Neurology, Philipps-Universität Marburg, Marburg, Germany; 3 Institute for Neurogenomics, Helmholtz Center for Health and Environment, Munich, Germany; 4 Department of Nuclear Medicine and Molecular Imaging, University Medical Center Groningen, University of Groningen, Groningen, The Netherlands

**Keywords:** Parkinson’s disease, Idiopathic REM sleep behavior disorder, ^18^F-FDG PET, fMRI, Biomarkers, Brain networks, Neuro-imaging

## Abstract

Parkinson’s disease (PD) commences several years before the onset of motor features. Pathophysiological understanding of the pre-clinical or early prodromal stages of PD are essential for the development of new therapeutic strategies. Two categories of patients are ideal to study the early disease stages. Idiopathic rapid eye movement sleep behavior disorder (iRBD) represents a well-known prodromal stage of PD in which pathology is presumed to have reached the lower brainstem. The majority of patients with iRBD will develop manifest PD within years to decades. Another category encompasses non-manifest mutation carriers, i.e. subjects without symptoms, but with a known mutation or genetic variant which gives an increased risk of developing PD. The speed of progression from preclinical or prodromal to full clinical stages varies among patients and cannot be reliably predicted on the individual level. Clinical trials will require inclusion of patients with a predictable conversion within a limited time window. Biomarkers are necessary that can confirm pre-motor PD status and can provide information regarding lead time and speed of progression. Neuroimaging changes occur early in the disease process and may provide such a biomarker. Studies have focused on radiotracer imaging of the dopaminergic nigrostriatal system, which can be assessed with dopamine transporter (DAT) single photon emission computed tomography (SPECT). Loss of DAT binding represents an effect of irreversible structural damage to the nigrostriatal system. This marker can be used to monitor disease progression and identify individuals at specific risk for phenoconversion. However, it is known that changes in neuronal activity precede structural changes. Functional neuro-imaging techniques, such as ^18^F-2-fluoro-2-deoxy-D-glucose Positron Emission Tomography (^18^F-FDG PET) and functional magnetic resonance imaging (fMRI), can be used to model the effects of disease on brain networks when combined with advanced analytical methods. Because these changes occur early in the disease process, functional imaging studies are of particular interest in prodromal PD diagnosis. In addition, fMRI and ^18^F-FDG PET may be able to predict a specific future phenotype in prodromal cohorts, which is not possible with DAT SPECT. The goal of the current review is to discuss the network-level brain changes in pre-motor PD.

## Introduction

Parkinson’s disease (PD) is a common neurodegenerative disorder which probably has a multifactorial disease etiology, consisting of environmental, genetic and epigenetic risk factors. The disease is associated with a sequential propagation of abnormal α-synuclein aggregates throughout the central and peripheral autonomic nervous system. It is unclear where alpha-synuclein pathology originates and how it spreads. A recent compelling hypothesis states that roughly two subtypes of PD exist (Borghammer and van den Berge 2019). A brain-first type, where α-synuclein initially arises in the brain with secondary spreading to the peripheral autonomic nervous system, and a body-first type, where the pathology originates in the enteric or peripheral autonomic nervous system and then spreads to the brain via the brainstem. This hypothesis is supported by recent in-vivo multimodality imaging studies of PD patients at different disease stages (Knudsen et al. 2018; Horsager et al. 2020).

The symptoms that PD patients experience depend on which neuronal systems are affected, and in which sequence. Currently, PD is definitively diagnosed when typical motor symptoms are present, as a result of degeneration of the presynaptic dopaminergic system (Postuma et al. 2015). These motor symptoms become apparent after 50–70% of nigrostriatal dopamine function has been lost (Berg et al. 2018; Kordower et al. 2013). By extrapolation, the disease process in the substantia nigra begins several years before the onset of motor symptoms (Hilker et al. 2005). In patients with the body-first subtype, the prodromal phase may be especially prolonged, and can be characterized by several non-motor symptoms, including constipation, hyposmia, sleep disorders (especially REM sleep behavior disorder), and depression, which are likely to be associated with neurotransmitter deficits other than dopamine.

**Fig. 1:**
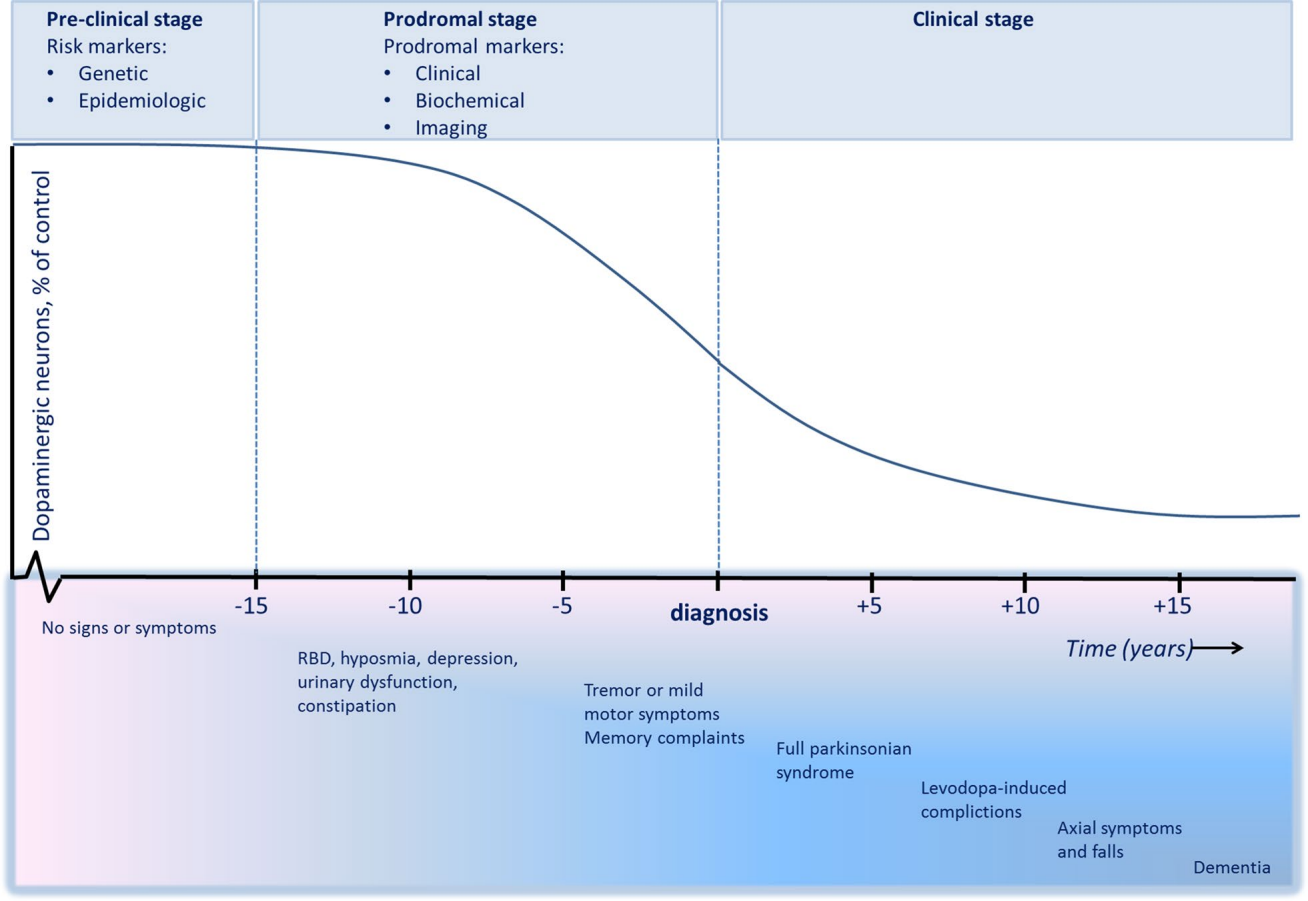
Schematic of preclinical, prodromal and clinical stages in PD. The y-axis shows the percentage of dopaminergic neurons in the substantia nigra. The exact course of dopaminergic attrition is unknown and is provided here schematically. The x-axis depicts the number of years before (left) and after (right) diagnosis. The exact duration of the preclinical and prodromal stages is unknown. The order of prodromal symptoms may vary between patients and are mentioned here in random order. The disease course depicted in this schematic only applies to patients with the so-called ‘body-first’ subtype of Parkinson’s disease

This implies that by the time PD is definitively diagnosed, disease progression is already advanced. Intervention at the stage of established PD may be too late in the pathological process for potential disease-modifying therapies to have any effect. In order to advance our understanding of the disease and develop therapeutic strategies, efforts are being made to further characterize the earliest stages of PD (Stern et al. 2012; Heinzel et al. 2019). In the preclinical stage, the neurodegenerative process has commenced, but there are no symptoms or signs. In the prodromal stage, symptoms and signs are present, but are yet insufficient to definitively diagnose PD (Fig. 1).

Individuals with a known genetic risk of Parkinson’s disease but without motor symptoms are suitable candidates to study the preclinical and prodromal stages of PD. Although patients with a specific gene mutation may be different from those with sporadic (‘idiopathic’) PD, genetic PD provides a model for studying disease onset and progression. Approximately 5–10% of all PD is caused by penetrant monogenes. These range from rare variants with very high penetrance, to genetic variants that are relatively common in the general population but exert only a modest effect on PD risk (Cherian and Divya 2020). A simplified overview of some of the genes implicated in PD is provided in Table 1. Mutations in LRRK2 and GBA genes are relevant in the context of studying pre-clinical or prodromal PD, because they are relatively frequent, give a clinical phenotype which is similar to sporadic (non-familial) PD, and have an age-dependent penetrance.

**Table 1:** Simplified overview of some of the genes implicated in Parkinson’s disease

Gene	Nomenclature (locus)	Known mutations/variants	Clinical features	Penetrance by age 80^*^
*Autosomal dominant forms of PD*				
SNCA	PARK1 and PARK 4	Missense mutations: A53T, A30P, E46K, G50D Duplications and triplications	YOPD, atypical and severe phenotypes depending on the specific mutation (i.e. triplications give a more severe phenotype)	probably high, > 90% for A53T, unknown for others
LRRK2	PARK8	G2019S: a missense mutation which is a frequent determinant of familiar and sporadic PD R1441G, Y1699C, I2020T	Classical (late-onset) PD	G2019S: 25–74%
GBA1	–	Mutations in GBA1 gene (NM_000157.3), also associated with Gaucher disease: N370S, S2716, L444P GBA1 variants (not associated with Gaucher disease)	Classical PD but with a slightly earlier onset age, severe motor impairment and higher prevalence of dementia and RBD	N370S, S2716: low risk, 7.6% L444P: high risk, 11–29.7%
*Autosomal recessive forms of PD*				
Parkin	PARK2		YOPD	100%
PINK1	PARK6		YOPD	100%
DJ-1	PARK7		YOPD	100%

*PD*  Parkinson’s disease, *SNCA*  α-synuclein, *LRRK2*  leucine-rich repeat kinase 2, *GBA*  glucocerebrosidase, *RBD*  REM sleep behavior disorder, *YOPD*  young onset PD, *PINK1*  PTEN induced putative kinase 1

*As determined by Heinzel et al. (2019)

GBA encodes glucocerebrosidase, and some mutations in the GBA gene cause the autosomal-recessive lysosomal storage disorder Gaucher’s disease in biallelic carriers. It is hypothesized that glucocerebrosidase plays a role in α-synuclein degradation. PD patients who carry GBA mutations and variants appear to have a more rapid progression of both motor and cognitive symptoms (Davis et al. 2016). GBA mutations and variants in PD have drawn attention because glucocerebrosidase provides a possible target for causative therapies (Greuel et al. 2020).

PD patients with LRRK2 mutations have a slower motor progression than idiopathic PD and are less likely to have cognitive impairment (Saunders-Pullman et al. 2018). An interesting finding is that in animal models, LRRK2-G2019S mutations cause a gain-of-abnormal function to striatal excitatory circuits during a critical early stage of development. It is thought that during this stage, a heightened activity of striatal spiny projection neurons permanently affects striatal circuit structure and function, possibly causing a risk of PD later in life (Matikainen-Ankney et al. 2016).

The prodromal disease stage can be investigated in individuals who have one or several non-motor symptoms that are known to precede the onset of typical motor symptoms in PD. Symptoms such as olfactory loss, constipation and depression are common in the general population and have low specificity and positive predictive values for conversion from probable prodromal PD to clinical PD. The strongest prodromal clinical marker thus far, is the presence of idiopathic rapid eye movement (REM) sleep behavior disorder (iRBD), a parasomnia that can be diagnosed with a polysomnography. Patients with iRBD fail to suppress muscle tone during the REM sleep stage, leading to dream enactment. Longitudinal studies have shown that > 80% of patients initially diagnosed with iRBD converted to clinical PD or dementia with Lewy bodies (DLB) in the following decades (Postuma et al. 2009, 2012; Iranzo et al. 2014, 2013; Schenck et al. 2013). DLB is also characterized by α-synuclein aggregates and overlaps with PD in terms of clinical characteristics and pathology. PD and DLB are often considered to be part of the same disease spectrum. Patients with iRBD may also develop multiple system atrophy (MSA), albeit much less frequently than PD or DLB. MSA is also an α-synucleinopathy, but is a distinct disease entity which should be seen separately from PD/DLB.

The positive predictive value of iRBD for conversion to clinical PD is high and exceeds even that of LRRK2 mutation carrier status. Prodromal individuals with iRBD are thought to have a ‘body-first’ subtype of PD, because they show marked autonomic damage before involvement of the dopaminergic system (Borghammer and Berge 2019). Patients with a premotor phase that includes iRBD usually develop a severe akinetic-rigid PD phenotype with prominent cognitive dysfunction (often dementia) (Fereshtehnejad and Postuma 2017).

The speed of progression from preclinical or prodromal to full clinical stages varies among patients and, at this time, cannot be reliably predicted on the individual level. Clinical trials will require inclusion of patients with a predictable conversion within a limited time window. Biomarkers are necessary that can confirm pre-motor PD status and can provide information regarding lead time and speed of progression. Imaging changes occur in the early phase of PD and are likely to become a standard part of early diagnosis. A combination of ^123^I-ioflupane single photon emission computed tomography (^123^I-FP-CIT SPECT or DAT SPECT) of the brain and ^123^I-metaiodobenzylguanidin (^123^I-MIBG) SPECT of the heart may be especially informative. A clearly abnormal ^123^I-FP-CIT SPECT scan reflects a presynaptic deficit in the nigrostriatal dopamine system and indicates imminent conversion to clinical PD. An abnormal ^123^I-MIBG SPECT scan reflects cardiac sympathetic denervation, indicating severe damage to the component of the autonomic peripheral nervous system (Heinzel et al. 2019).

In large iRBD cohorts, approximately 50% of patients have nigrostriatal dopamine innervation within normal limits (Bauckneht et al. 2018). In cases with an abnormal ^123^I-FP-CIT SPECT scan, the dopaminergic deficit is usually less severe compared to established PD. If a patient has a reduction of more than 25% of normal in putaminal DAT binding, phenoconversion within three years is likely (Bauckneht et al. 2018; Iranzo et al. 2017). ^123^I-MIBG SPECT is nearly always abnormal in iRBD (94.3% of cases) (Borghammer and Berge 2019). This means that patients with iRBD, who can be considered to have prodromal (i.e. pre-motor) PD, almost always have cardiac sympathetic denervation, but their nigrostriatal dopamine system can still be relatively intact.

Limited data exists on ^123^I-FP-CIT and ^123^I-MIBG SPECT imaging studies in asymptomatic PD gene mutation carriers (for review see (Matarazzo et al. 2018; Varrone and Pellecchia 2018)). A 4-year longitudinal study in 32 asymptomatic LRRK2 mutation carriers showed that a lower striatal DAT binding at baseline predicted subsequent conversion to clinical PD (which was 12%) (Sierra et al. 2017). To the best of our knowledge, ^123^I-MIBG SPECT has not been evaluated extensively in pre-symptomatic carriers of PD associated genes. In patients with manifest LRRK2 PD, approximately half have an abnormal ^123^I-MIBG SPECT. ^123^I-MIBG SPECT is pathological in almost all patients with manifest PD due to GBA or SNCA gene mutations (Borghammer and Berge 2019).

^123^I-FP-CIT SPECT reflects structural brain changes as a result of pathology in the nigrostriatal dopaminergic system. However, it is known that changes in neuronal activity precede such structural changes. The main principle of functional neuro-imaging techniques is that localized changes in neuronal activity can be mapped by measuring changes in energy metabolism or hemodynamics, which reflect the underlying cellular events. The radiotracer ^18^F-2-fluoro-2-deoxy-D-glucose (^18^F-FDG) is a glucose analogue, and allows assessment of cerebral glucose metabolism in-vivo with positron emission tomography (PET). Functional magnetic resonance imaging (fMRI) is based on the blood oxygen level dependent (BOLD) response to neuronal activity. When combined with advanced analytical methods, ^18^F-FDG PET and fMRI can be used to model the effects of disease on the disintegration of normal brain networks. Because these changes occur early in the disease process, functional imaging studies are of particular interest in prodromal and preclinical PD diagnosis. In addition, fMRI and ^18^F-FDG PET may be able to predict a specific future phenotype in prodromal cohorts, which is not possible with ^123^I-FP-CIT SPECT and ^123^I-MIBG SPECT alone. In this review, we discuss resting-state functional neuro-imaging studies in iRBD and non-manifest mutation carriers.

## Univariate cerebral metabolism and blood flow studies in iRBD

In PD, abnormal accumulation of α-synuclein in neurons impairs synaptic signaling, causing disintegration of specific neural networks. Neuro-imaging with ^18^F-FDG PET can capture synaptic dysfunction in vivo. The radiotracer ^18^F-FDG provides an index for the cerebral metabolic rate of glucose, which is strongly associated with neuronal activity and synaptic integrity (Reivich et al. 1979). Brain regions with altered ^18^F-FDG uptake can be identified with univariate group-comparisons (patients versus controls) using Statistical Parametric Mapping (SPM). PD is typically characterized by relatively increased metabolism in the putamen, thalamus, cerebellum, pons and sensorimotor cortex, and relatively decreased metabolism in the lateral frontal and parieto-occipital areas (Peng et al. 2014). The pattern in DLB is similar but with more extensive hypometabolism of the occipital cortex and posterior parietotemporal areas (Yousaf et al. 2019).

Studies evaluating brain metabolic differences between controls and iRBD have shown some overlap with findings in PD and DLB but also show inconsistencies. Most studies found relative hypometabolism of the occipital cortex compared with controls (Fujishiro et al. 2010, 2013; Ge et al. 2015; Carli et al. 2020a), but others only reported decreased metabolism in parieto-temporal regions (Caselli et al. 2006; Liguori et al. 2019). Hypermetabolic areas were also variable, including brainstem, hippocampus, frontal cortex, supplementary motor area, and putamen (Ge et al. 2015; Carli et al. 2020a; Liguori et al. 2019).

To investigate individual variation in brain metabolism further, Carli et al. performed an SPM-based single-subject procedure, in which the ^18^F-FDG PET scan of each individual iRBD patient was compared to a large database of healthy controls (Carli et al. 2020a). The resulting single-subject SPM maps were evaluated by expert raters blinded to the diagnosis. Several distinct hypometabolism patterns were identified: selective occipital hypometabolism (n = 5), occipitoparietal hypometabolism (n = 13), and occipital and cerebellar hypometabolism (n = 13). There was one case with isolated cerebellar hypometabolism, of which the authors suspected an etiology of prodromal MSA. Individual hypermetabolism patterns were also heterogeneous, variably including (para)hippocampus, amygdala, caudate, putamen/pallidum and cerebellum. Five cases had a completely normal ^18^F-FDG PET scan. The authors suggest that iRBD is an intrinsic heterogeneous condition. One explanation may be that in these cross-sectional studies, each individual is scanned at a different time point on his/her course of disease progression towards manifest PD/DLB (or MSA). In addition, patients may follow distinct trajectories towards their final diagnoses in which different neuronal networks are affected over time, perhaps due to differential vulnerability of cell populations or certain compensatory effects.

One longitudinal study analyzed regional ^18^F-FDG PET brain changes in 20 iRBD patients at baseline, and after 2 and 4 years (Kim et al. 2021). Significant metabolic increases over time were observed in the putamen (left and right) and bilateral cuneus and lingual gyrus across all time points. Metabolism decreased significantly in the bilateral premotor cortex, supplementary motor area, and superior frontal gyrus. During a median of 5.4 years, 8 iRBD patients converted to manifest PD (n = 3), DLB (n = 4) and MSA (n = 1). Converters had greater metabolic increases in the bilateral putamen. PD and DLB converters showed decreases in metabolism in the premotor cortex, superior frontal gyrus and supplementary motor area over time, whereas the MSA-P converter showed increases in these regions. Changes in the occipital cortex were inconsistent.

Three longitudinal studies have been performed in iRBD which used ^99m^Tc-Ethylene Cysteinate Dimer (^99m^Tc-ECD) SPECT to quantify regional cerebral blood flow (Dang-Vu et al. 2012; Sakurai et al. 2014; Baril et al. 2020). In normal conditions, glucose metabolism and cerebral blood flow are closely coupled (Fox and Raichle 1986; Fox et al. 1988). Similar disease patterns have been obtained with ^18^F-FDG PET and ^99m^Tc-ECD SPECT (Peng et al. 2014). Cerebral blood flow studies in iRBD have also yielded similarly heterogeneous results as those using ^18^F-FDG PET (Caselli et al. 2006; Sakurai et al. 2014; Mazza et al. 2006; Vendette et al. 2011; Hanyu et al. 2011).

Dang-vu et al. studied the association between regional cerebral blood flow changes in 20 iRBD patients at baseline and subsequent conversion to PD or DLB over the course of three years of clinical follow-up. Ultimately five iRBD patients converted to PD, and five converted to DLB. Hippocampal perfusion was increased in converters compared to non-converters, and was significantly correlated with motor and color vision scores (Dang-Vu et al. 2012). No clear differences in cerebral blood flow were reported between iRBD patients who converted to PD (n = 5) and those who converted to DLB (n = 5).

Sakurai et al. performed blood flow SPECT in nine iRBD patients at baseline and after approximately 2 years (Sakurai et al. 2014). Three-dimensional stereotactic surface projections (3D-SSP (Minoshima et al. 1995)) were created for each scan and compared to data from 18 controls. Overall, patients had lower cerebral blood flow in the bilateral parietotemporal and occipital areas. Although these nine patients did not phenoconvert during the study, there was a progressive decrease in perfusion of the posterior cingulate cortex.

Baril et al. performed ^99m^Tc-HMPAO SPECT in 37 iRBD patients at baseline and after a median of 12 months (Baril et al. 2020). At baseline, they found relatively decreased perfusion of the anterior frontal and lateral parietotemporal cortex compared to healthy controls (n = 23). Interestingly, perfusion of these areas increased to normal levels at follow-up. Areas of relatively increased metabolism were not clearly identified. It must be noted that cerebellum and brainstem were excluded from the analysis. During follow-up (1–5 years after imaging), 5 developed parkinsonian features, and 4 subjects were diagnosed with DLB; subgroup analyses in converters were not performed. The authors suggest that this renormalization of the perfusion pattern over time reflects compensatory mechanisms of the brain to cope with alpha-synuclein pathology.

## Metabolic connectivity networks in PD

Regions with correlated metabolic activity are considered to be functionally interconnected. Therefore, ^18^F-FDG PET can visualize the effect of local pathology on brain networks, when combined with computational algorithms based on pattern recognition and machine learning. Spatial covariance analysis has been used to identify relevant disease-related patterns in ^18^F-FDG PET data by taking into account the relationship (covariance) between voxels across subjects. This way, abnormal brain networks typical for the disease can be identified. Moreover, once a disease-related pattern is uncovered, the degree to which a single patient’s ^18^F-FDG PET scan resembles the pattern can be determined. This degree of pattern expression is reflected by a single numeric value (the subject score), usually represented by a z-score. A healthy control group typically has a mean z-score of zero, and a high z-score reflects more extreme changes of cerebral metabolism in the direction of the pattern.

By quantifying disease-related pattern expression on a scan-by-scan basis, this technique allows objective assessment of disease activity in individual subjects (Eidelberg 2009; Meles et al. 2021). With spatial covariance analysis, a metabolic network has been identified in PD, referred to as the PD related pattern (PDRP). The PDRP has been thoroughly validated and identified in multiple populations (Schindlbeck and Eidelberg 2018; Meles et al. 2019), and resembles the regional changes found in univariate SPM studies of PD (Peng et al. 2014; Ma et al. 2009).

The PDRP is consistently characterized by relative hypermetabolism in the thalamus, putamen/pallidum, pons, cerebellum and motor cortex, and relatively decreased metabolism in the lateral premotor and parieto-occipital cortex. This topography reflects synaptic dysfunction in sporadic PD measured in vivo, and provides important insights into the pathophysiology of PD. The relatively hyperactive (‘red’) regions in the PDRP (i.e. thalamus, putamen/pallidum, pons, cerebellum and motor cortex) are thought to be central in brain network dysfunction in PD. These structures comprise important nodes in widely distributed cortico-striatal-pallido-thalamo-cortical (CSPTC) circuits, which are modulated by dopaminergic input to the striatum (DeLong and Wichmann 2007; Alexander et al. 1986; Rodriguez-Oroz et al. 2009). The fact that these regions are hyper- rather than hypometabolic, may be explained by alterations in neuronal firing rates and firing patterns in networks of cortico-basal ganglia neurons, caused, at least in part, by focal loss of dopaminergic drive to the striatum (Rivlin-Etzion et al. 2006).

Neuronal firing rates in the subthalamic nucleus (STN) and globus pallidus pars interna (GPi) are increased in PD. Positive correlations have been found between PDRP subject scores obtained from pre-operative ^18^F-FDG PET data of PD patients undergoing deep-brain stimulation and intra-operatively recorded STN and GPi firing rates (Lin et al. 2008; Eidelberg et al. 1997). On a regional level, positive correlations were especially apparent between STN firing rates and putamen, globus pallidus, and primary motor cortex (Lin et al. 2008).

In addition to increased firing rates, the pattern of discharge is altered in the STN and GPi, with neighboring neurons shifting from spontaneous firing to synchronized oscillations (Oswal et al. 2013; Wichmann and DeLong 2003). Increased synchronicity of neural firing rates is associated with an increased functional coupling of neurons. In terms of network structure, networks that facilitate synchronization are associated with a small-world configuration. In small-world networks, there is a shortened communication distance between regions and increased connectivity between neighboring neurons. These properties normally serve to optimize the efficiency of information transfer in a network, at reduced energetic cost (Reijneveld et al. 2007). Excessive synchronization of local neural activity may be detrimental to circuit performance (Brittain and Brown 2014).

Spatial covariance patterns, such as the PDRP, are whole-brain maps in which each voxel is assigned a value according to its relative importance in the pattern (and by extension, its relative importance in determining the subject score). It discloses no information on connections between major regions. In other words, the PDRP represents changes in brain function but not network structure. To better understand the structural organization of hyper- and hypometabolic regions in the PD network, Ko et al. applied graph theoretical analysis to ^18^F-FDG PET scans of PD patients (Ko et al. 2018). Graph theory independently identified the hypermetabolic (‘red’) PDRP regions as the core of an abnormal PD network with exaggerated small world properties. There are pathological links between the ‘red’ regions, which incur a high energetic cost. In turn, the more weakly connected peripheral nodes in the graph corresponded to the hypometabolic cortical regions of the PDRP.

It appears that regions with relatively increased metabolism in the PDRP are especially relevant to the early stages of the disease. In a longitudinal study, 15 early stage (unilaterally affected) PD patients were scanned three times over a period of four years with ^18^F-FDG PET and DAT SPECT (Huang et al. 2007). Disease progression was associated with increasing metabolism in the STN, GPi, dorsal pons and primary motor cortex; and with declining metabolism in the prefrontal and inferior parietal regions. PDRP expression increased progressively over time, and was correlated with concurrent declines in striatal DAT binding and increases in motor ratings (Huang et al. 2007). The rate of progression was relatively greater in the regions with relatively increased metabolism (the red network core), compared to the regions with relatively decreased metabolism (peripheral nodes). Changes in this metabolically active PDRP core, which involves regions connected to the substantia nigra, basal forebrain and para-limbic structures, in fact appear to precede those in the (blue) peripheral nodes (Ma et al. 2009).

One study examined PDRP expression in manifest PD patients with LRRK2-2019S mutations (PD-LRRK2) and GBA1 variants (PD-GBA) (Schindlbeck et al. 2020). Both PD-LRRK2 and PD-GBA groups had PDRP subject scores that were higher compared to controls. PDRP subject scores in PD-GBA were higher than in idiopathic PD matched for age, symptom duration and off-medication motor disability ratings. This possibly reflects the more progressive nature of PD-GBA. PDRP scores in PD-LRRK2 were on average slightly lower, reflecting a more benign disease course.

Analogous to the study by Ko et al. in idiopathic PD (Ko et al. 2018), a graph theoretical analysis was applied to investigate the effects of genotype on the organization of the PD network (Schindlbeck et al. 2020). Both PD-LRRK2 and PD-GBA patients exhibited abnormal increases in network connectivity that were not present in sporadic PD. The PD-LRRK2 group showed increased functional connectivity within the red core of the PDRP network, especially within cerebello-thalamo-putamen pathways. This reorganization may mirror findings of altered striatal circuit structure and function found in animal models during development. In PD-GBA, functional connections were gained outside the ‘red’ core, in the peripheral nodes, involving cortico-cortical pathways, consistent with a more aggressive disease course and cognitive dysfunction seen in PD-GBA. To the best of our knowledge, PDRP network expression and structure has not been investigated in pre- or asymptomatic gene carriers. It is therefore difficult to determine if these findings of altered connectivity are compensatory or pathogenic.

## PDRP network expression in iRBD

PDRP expression can be detected in PD patients before the onset of motor features. One study calculated PDRP expression in each hemisphere instead of in the whole brain, and found that PDRP expression was already higher compared with healthy controls in the presymptomatic hemisphere (i.e., ipsilateral to the symptomatic body side) of patients with early-stage PD with unilateral motor involvement (Tang et al. 2010). This network change anteceded the onset of motor signs on the opposite body side by approximately 2 years. In the pre-symptomatic hemisphere, reductions in putamen DAT binding were inversely related to concurrent increases in metabolic activity in this structure. In contrast, hypermetabolism in the putamen of the symptomatic hemisphere remained stable across all time points. The authors suggest that the increases in metabolic activity in the putamen are a functional response to nigrostriatal dopamine depletion beyond a specific threshold, and that the onset of motor features is closely linked to putamen hyperactivity.

**Fig. 2:**
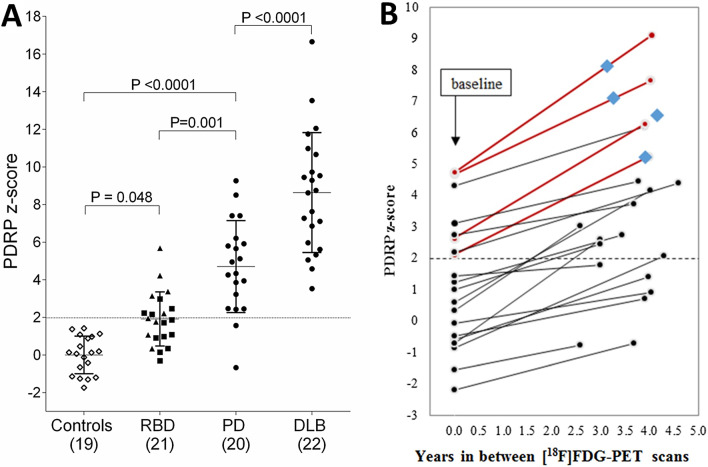
**A** PDRP z-scores across groups. PDRP expression was calculated in all groups and z-transformed to the healthy controls. PDRP expression z-scores were compared across groups with a one-way analysis of variance. Post-hoc comparisons were Bonferroni-corrected. Triangles indicate patients with an abnormal DAT scan, squares indicate patients with a normal DAT scan. From: (Meles et al. 2017). The dashed line indicates z = 1.98, scores above this line are considered supra-threshold (see (Kogan et al. 2020)). **B** PDRP expression z-score changes between baseline and follow-up ^18^F-FDG PET imaging in 20 iRBD patients. PDRP expression increased in all subjects. Four subjects (indicated by Burgundy lines), all with baseline significant PDRP z-scores, phenoconverted to clinical PD during the study. Diamonds denote point of clinical phenoconversion. From: (Kogan et al. 2020)

Several studies have shown that expression of the PDRP is elevated in iRBD patients compared to controls, and is on average lower compared to established PD (Holtbernd et al. 2014; Wu et al. 2014; Meles et al. 2017; Yoon et al. 2019; Huang et al. 2020) (Fig. 2A).

In a cohort of 17 patients, high baseline PDRP expression (a PDRP subject score of > 1) on brain perfusion imaging (^99m^Tc-ECD SPECT) was more likely in iRBD patients (n = 8) who developed PD or DLB 4.6 ± 2.5 years after getting scanned. In contrast, 3 iRBD patients who developed MSA 2–4 years later did not express the PDRP at baseline. These findings indicate that network expression has potential as a marker for phenoconversion and may have additional value in predicting which phenotype a patient with idiopathic RBD will develop (Holtbernd et al. 2014).

Kogan et al. performed ^18^F-FDG PET twice in 20 iRBD subjects, approximately 4 years apart, and calculated PDRP expression at each time point (Kogan et al. 2020) (Fig. 2B). PDRP subject scores showed consistent, significant increases between baseline and follow-up imaging in all iRBD subjects, which corresponded to changes in motor function. Four subjects (20%) with high PDRP z-scores converted to manifest PD at follow-up.

Kim et al. studied 20 iRBD patients with ^18^F-FDG PET at baseline, 2 and 4 years. During a median of 5.4 years, 8 iRBD patients converted to manifest PD (n = 3), DLB (n = 4) and MSA (n = 1). There was a trend of higher PDRP z-scores in converters compared to non-converters, but this was not extensively analyzed, as this was not the focus of the study (Kim et al. 2021).

## Metabolic brain networks that characterize the iRBD state

Three cross-sectional studies have identified an abnormal disease-related pattern in ^18^F-FDG PET scans of iRBD patients using spatial covariance analysis (i.e. the RBD-related pattern, iRBDRP), with similar inconsistencies as was the case in univariate ^18^F-FDG PET studies. Wu et al. describe an iRBDRP which is characterized by relative hypermetabolism of the pons, thalamus, medial frontal and sensorimotor areas, hippocampus, supramarginal and inferior temporal gyri, and posterior cerebellum, and relative hypometabolism in the occipital and superior temporal regions. This iRBDRP was significantly expressed in iRBD and patients with early, unilateral PD, but subject scores were lower in patients with more advanced PD, indicating that the metabolic changes from iRBD to advanced PD do not follow one pattern. The authors suggest that the iRBDRP topography breaks down with disease progression (Wu et al. 2014).

A very similar iRBDRP topography was found in a Korean cohort (Yoon et al. 2019). Kim et al. subsequently investigated iRBDRP expression in 20 iRBD patients at baseline, and after 2 and 4 years of follow-up (Kim et al. 2021). Expression of the iRBDRP decreased over time in patients, supporting the hypothesis of Wu et al. The  iRBDRP breakdown was explained by attenuation of relative hypermetabolism of the frontal cortex over time.

**Fig. 3:**
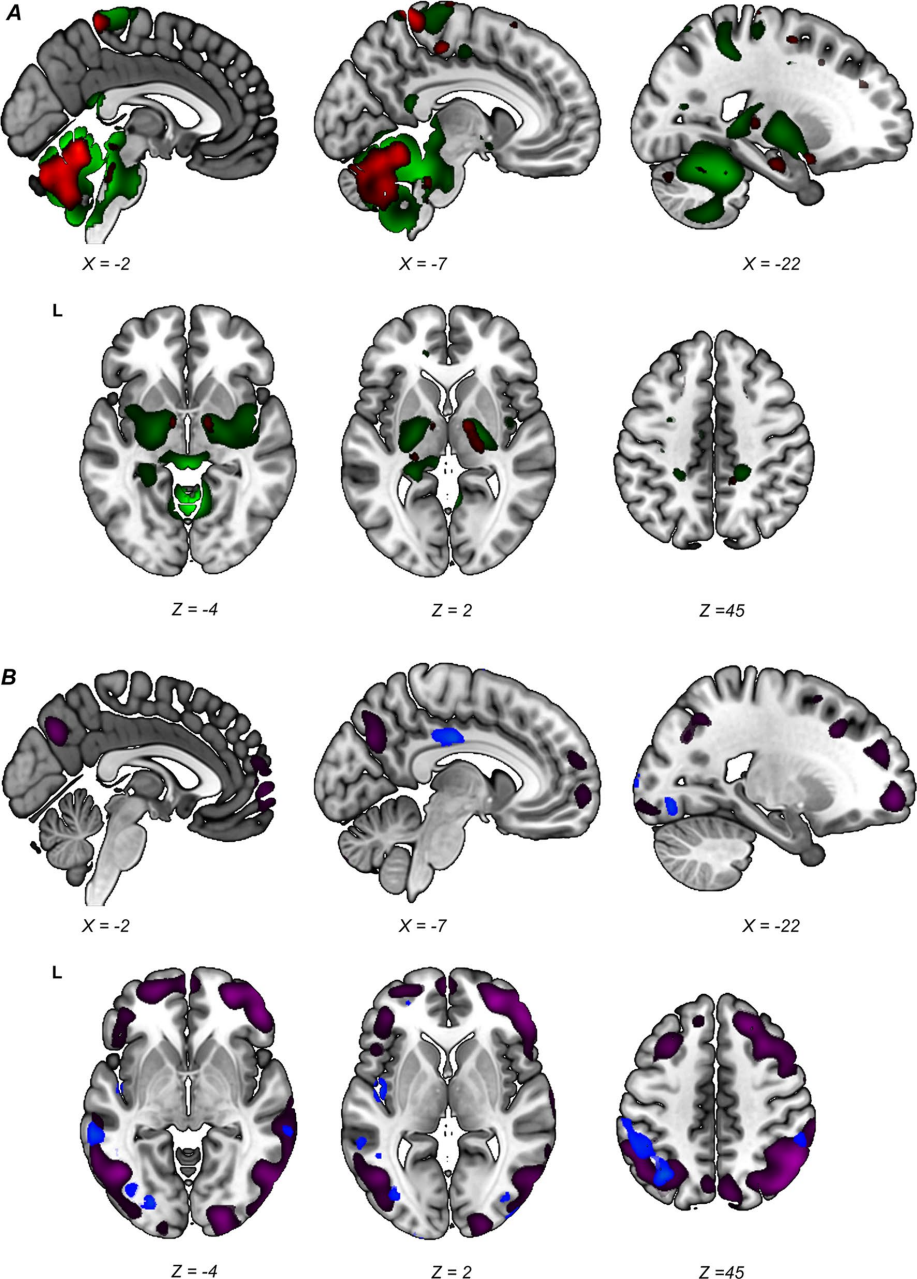
Stable regions in iRBDRP and PDRP overlap. Stable voxels (90% confidence interval not straddling zero after bootstrap resampling) of iRBDRP and PDRP are overlaid on T1 MRI template. **A** Stable, relatively hypermetabolic regions of PDRP (green) and iRBDRP (red). **B** Stable, relatively hypometabolic regions of PDRP (purple) and iRBDRP (blue). L = left. Coordinates in axial (Z) and sagittal (X) planes are in Montreal Neurologic Institute standard space. From: (Meles et al. 2018)

In contrast to the aforementioned studies, Meles et al. identified an iRBDRP which was expressed in both early and more advanced PD patients (Meles et al. 2018). This iRBDRP showed striking overlap with the PDRP (Fig. 3). In comparison with the other iRBDRPs, cerebellar hypermetabolism was a more prominent feature, and frontal hypermetabolism and occipital hypometabolism were less pronounced. The fact that these iRBDRP topographies are not as universal as the PDRP is perhaps unsurprising given that the metabolic changes at the early stages of neurodegeneration are subtle, subject to both ongoing neurodegeneration and functional compensation, and heterogeneity of patient samples.

## Neurotransmitter networks in iRBD

PDRP expression is inversely correlated to dopaminergic decline in PD. This correlation is typically modest in magnitude (Niethammer and Eidelberg 2012; Holtbernd et al. 2015). In a cross-sectional combined ^18^F-FDG PET and DAT SPECT study in 21 iRBD patients, PDRP subject scores were not significantly correlated with loss of DAT binding, although a trend was observed (Meles et al. 2017). In a larger study (n = 37), PDRP expression z-scores correlated significantly with DAT binding in a subgroup of iRBD patients with abnormal DAT binding (n = 18), but not in subjects with normal DAT binding (n = 19) (Huang et al. 2020). In that study, PDRP z-scores were also calculated in each cerebral hemisphere separately and were found to be abnormally elevated in both hemispheres. A recent study in PD has also shown that PDRP expression remains symmetric across hemispheres even in later PD stages (Tang et al. 2020). This contrasts the asymmetric decline of striatal dopaminergic integrity. Taken together, it is likely that the PDRP reflects more complex neurodegenerative processes in which, in addition to derangements in the dopaminergic system, other monoaminergic deficiencies may also play a role.

Carli et al. aimed to investigate the derangement in three neurotransmitter systems by applying a region-of-interest (ROI)-based network analysis to ^18^F-FDG PET data of subjects with iRBD (n = 34), idiopathic PD (n = 29), DLB (n = 30) and 50 healthy controls (Carli et al. 2020b). For each neurotransmitter system (nigro-striatal-cortical dopaminergic, noradrenergic, cholinergic), specific ROIs were chosen that were likely involved in those particular systems. Connectivity analyses were performed on ROI-based ^18^F-FDG uptake within each network and across groups. As expected, the nigro-striato-cortical dopaminergic network was nearly intact in iRBD, but affected in PD and DLB. The noradrenergic system was almost equally affected in all three groups, whereas derangements in the cholinergic system were limited in PD but more extensive in iRBD and DLB, especially in the thalamus.

Several radiotracers are available to investigate specific neurotransmitter systems in vivo, but literature in iRBD is scarce. The noradrenergic locus coeruleus (LC), which has a long trajectory in the pons, is affected early and extensively in PD, and is associated with RBD and a variety of other non-motor symptoms. The LC provides the sole source of norepinephrine to the neocortex, hippocampus, cerebellum, and thalamus, and also exerts control over other nuclei, such as the substantia nigra and the raphe nuclei. The function of cortical projections from the LC can be visualized using the noradrenaline transporter PET ligand ^11^C-MeNER. Reduced ^11^C-MeNER binding was found in the sensorimotor cortex in iRBD patients (n = 17) and in PD patients with RBD (n = 16) compared with controls. Loss of ^11^C-MeNER binding in the thalamus correlated with loss of putaminal 18F-dopa binding, suggesting that the noradrenergic and dopaminergic neurotransmitter systems degenerate in parallel in iRBD (Andersen et al. 2020).

Two studies have examined the cholinergic system in iRBD. One study applied ^11^C-donepezil PET, an in-vivo marker of acetylcholinesterase (AChE) levels, in 17 iRBD patients and 9 controls. Significant reductions in ^11^C-donepezil levels were found in the bilateral superior temporal cortex, occipital cortex, cingulate cortex and dorsolateral prefrontal cortex (Gersel Stokholm et al. 2020). This is in accordance with previous studies in patients with manifest PD and DLB (Kogan et al. 2019).

Bedard et al. used the radiotracer ^18^F-fluoroethoxybenzovesamicol (FEOBV) in a small study of 5 iRBD subjects and 5 healthy controls (Bedard et al. 2019). FEOBV binds to the vesicular acetylcholine transporter, and can measure the density of cholinergic nerve terminals. Interestingly, this study did not find any cortical cholinergic reductions. Rather, FEOBV binding was increased in iRBD compared to controls in the brainstem, thalamus, cerebellum, paracentral cortex, anterior cingulate and prefrontal cortex. It was suggested that degeneration of the cholinergic system starts in the prodromal phase and that cholinergic nerve terminals initially increase as a compensatory mechanism, and later decrease with progressive neurodegeneration.

## fMRI studies in non-manifest PD gene carriers

Energy consumption (i.e. glucose metabolism) is tightly linked to blood flow (neurovascular coupling), but not to oxygen utilization. Functional magnetic resonance imaging (fMRI) is based on the blood oxygen level dependent (BOLD) response, which is measured by the change in the magnetic field due to altered ratios in oxygenated and deoxygenated blood in response to varying neuronal activity (Fox and Raichle 2007). The BOLD signal reflects the net effect of oxygen consumption (which decreases BOLD) and blood flow increase (which increases BOLD) in response to neuronal activity (Hall et al. 2016). Resting-state fMRI paradigms can give information about task-invariant aspects of brain function. A few resting-state fMRI studies have been performed in pre-symptomatic PD gene carriers (for review see Thaler (2018)). Here we discuss the most relevant ones.

Two studies in non-manifest LRRK2 mutation carriers have shown that altered connectivity in motor networks precedes manifest PD. Helmich et al. studied 37 asymptomatic LRRK2 G2019S mutation carriers and 32 matched, asymptomatic non-carriers (controls) with resting-state fMRI and a seed-based analysis focused on four striatal sub regions (dorsoposterior putamen, ventroposterior putamen, dorsoanterior putamen and caudate). In controls, there was a strong connectivity between the dorsoposterior putamen and the parietal cortex. In non-manifest mutation carriers, this connectivity had shifted to the ventroanterior part of the putamen, which is less affected by dopaminergic depletion. This shift increased with age in mutation carriers, but not in controls. A similar connectivity shift was seen in idiopathic PD. The authors concluded that these changes may reflect premotor basal ganglia dysfunction or circuit-level compensatory changes (Helmich et al. 2015). A second rsfMRI study assessing 18 LRRK2 non-manifest mutation carriers (13 G2019S, 3 R1441G and 2 R1441C) and 18 healthy non-carriers (controls), also found decreased connectivity between the caudal motor part of the striatum and the parietal cortex in non-manifest carriers. These subjects also showed increased functional connectivity between the substantia nigra and the occipital cortex (Vilas et al. 2016).

Jacob et al. investigated 44 non-manifest G2019S LRRK2 mutation carriers, and 41 non-carriers (controls) with rsfMRI to assess a motor network and several non-motor networks (the default mode, salience and dorsal attention networks) using a graph-theory based analysis method (Jacob et al. 2019). Interestingly, no significant differences were found between the two groups in the organization of the motor network. Yet, non-manifest carriers demonstrated reduced connectivity and nodal influence in all three non-motor networks compared with controls (especially in the salience network), with no increased connectivity findings. The authors conclude that cognitive networks might be affected before the motor network among “at risk” populations for future development of PD.

## fMRI studies in iRBD

Two seed-based (region-to-region and seed-to-voxel) rsfMRI studies have shown reduced functional connectivity within basal ganglia networks (Ellmore et al. 2013; Dayan and Browner 2017). Functional connectivity between the putamen and substantia nigra was reduced, but nonetheless higher in iRBD than PD, indicating a continuous spectrum of decline in functional connectivity (Ellmore et al. 2013). Another study revealed widespread aberrant functional connectivity within the basal ganglia network in iRBD using independent component analysis (Rolinski et al. 2016).

Byun et al. studied thalamo-cortical functional connectivity in 37 iRBD patients and 15 healthy controls using a seed-based approach. They found increased functional connectivity between the left thalamus and occipital regions (Byun et al. 2020). The authors suggest that this reflects a compensatory mechanism, as connectivity in posterior brain regions is progressively lost in patients with PD, especially in those patients with cognitive impairment (Olde Dubbelink et al. 2014). Congruent with findings in PD, a hypothesis-free, whole-brain graph-analysis based study of rsfMRI data disclosed dysfunction in posterior brain networks in iRBD (n = 20) compared with controls (n = 27). Reduced functional connectivity between associative regions of the temporal and parietal regions correlated with a lower cognitive processing speed (Campabadal et al. 2020). Interestingly, these abnormal findings had a left-hemisphere predominance (most iRBD patients were right-handed).

Li et al. aimed to investigate changes in functional connectivity within the central autonomic network (iRBD n = 32, controls n = 33). They selected ROIs as seeds for the functional connectivity analyses, based on a-priori assumptions of regions that may be regarded as central autonomic structures (brainstem, hypothalamus, amygdala, anterior cingulate and the insula). Compared with controls, functional connectivity was reduced between the brainstem and the cerebellum, temporal lobe and anterior cingulate. These changes were correlated with the severity of autonomic symptoms in patients. That said, the connectivity changes that were found may not be specific to autonomic systems (Li et al. 2020).

## Multi-modality imaging in prodromal PD

It is currently unknown when in the disease course brain networks in PD become abnormal. In the absence of a (readily available) α-synuclein tracer, multi-modality imaging is currently the only way to further investigate this topic in vivo. Knudsen et al. combined several imaging techniques in healthy controls, iRBD and PD to assess Braak stages I-III (Knudsen et al. 2018). Braak stage I was assessed with ^11^C-donepezil PET–CT (cholinergic innervation of the gut) and ^123^I-metaiodobenzylguanidine (MIBG) SPECT (noradrenergic innervation of the heart). These two imaging studies can be used to determine the integrity of the dorsal motor nucleus of the vagus nerve in the medulla. Integrity of the pontine locus coeruleus (stage II) was studied with neuro-melanin sensitive MRI and ^11^C-methylreboxetine (MeNER) PET, a noradrenergic tracer. Presynaptic dopaminergic imaging (^18^F-Dopa PET) was done to characterize stage III. The authors showed that iRBD patients have abnormal studies in Braak stages I and II, similar to PD patients. Only approximately 30% of iRBD patients had abnormal ^18^F-Dopa PET scans, in contrast to (by definition) 100% of the PD patients. There was a striking separation of controls versus iRBD/PD on ^123^I-MIBG. This suggests that any patient with iRBD who has abnormal findings on ^123^I-MIBG has an α-synucleinopathy. To date, functional neuroimaging studies have not been performed in such an extensive multi-modality imaging context.

## Concluding remarks and future perspectives

Functional neuroimaging studies have revealed brain alterations in prodromal PD, that mirror those found in PD and DLB. Most data stem from studies in iRBD. The inconsistencies between studies have been described in this review and are likely the result of differences in modalities and analytical methods, but also heterogeneity in patient samples. Studies in preclinical stages (i.e. pre-maninfest mutation carries) are scarce. Because of their rarity, studies sometimes clump patients with different mutations together in one group. In iRBD, patients at different stages of neurodegeneration are combined in one group and cross-sectionally compared with controls. Multi-modality imaging is necessary to make inferences about the individual’s stage in the neurodegenerative process. Longitudinal follow-up is also pertinent in both categories. Only when patients are followed longitudinally and the neuroimaging metric under investigation is performed more than once over time, can circuit-level changes be interpreted as compensatory and pathologic. This will require longitudinal studies in large samples, with confirmation of additional biomarkers. Such studies are currently lacking. Progress is being made in the realm of PDRP network expression. A study with sequential ^18^F-FDG PET studies in an iRBD cohort enriched with DAT SPECT and ^123^I-MIBG SPECT is currently underway, but will take time to reach sufficient numbers and follow-up. Another topic for future studies may be the genetic risk factors in iRBD. Most neuroimaging studies in iRBD are performed without genetic testing, and especially in smaller cohorts, results could potentially be confounded by the genetic risk of included patients.

## Data Availability

Not applicable.

## Abbreviations

BOLD: Blood oxygen level dependent
CSPTC: Cortico-striatal-pallido-thalamo-cortical
DAT: Dopamine transporter
DLB: Dementia with lewy bodies
^18^F-FDG: ^18^F-2-fluoro-2-deoxy-d-glucose
fMRI: Functional magnetic resonance imaging
GBA: Glucocerebrosidase
GPi: Globus pallidus pars interna
^123^I-FP-CIT: ^123^I-ioflupane
^123^I-MIBG: ^123^I-metaiodobenzylguanidin
iRBD: Idiopathic rapid eye movement sleep behavior disorder
iRBDRP: Idiopathic rapid eye movement sleep behavior disorder related pattern
LC: Locus coeruleus
LRRK2: Leucine-rich repeat kinase 2
MSA: Multiple system atrophy
PD: Parkinson’s disease
PDRP: Parkinson’s disease related pattern
PET: Positron emission tomography
PINK1: PTEN induced putative kinase 1
REM: Rapid eye movement
ROI: Region-of-interest
SNCA: α-Synuclein
SPECT: Single photon emission computed tomography
SPM: Statistical parametric mapping
STN: Subthalamic nucleus
^99m^Tc-ECD: ^99M^Tc-ethylene cysteinate dimer
YOPD: Young onset Parkinson’s disease
